# Autosomal recessive polycystic kidney disease (ARPKD) in a Nigerian newborn: a case report

**DOI:** 10.11604/pamj.2018.30.172.15202

**Published:** 2018-06-25

**Authors:** Olufunke Bolaji, Olagoke Erinomo, Olufunmilayo Adebara, Julia Okolugbo, Bartholomew Onumajuru, Taiwo Akanni, Olusegun Adebami

**Affiliations:** 1 Department of Paediatrics and Child Health, College of Medicine, Afe Babalola Univerisity, Ado-Ekiti, Nigeria and Federal Teaching Hospital, Ido-Ekiti, Nigeria; 2 Department of Morbid Anatomy and Histopathology, Federal Teaching Hospital, Ido-Ekiti, Nigeria; 3 Department of Paediatrics and Child Health, Federal Teaching Hospital, Ido-Ekiti, Nigeria; 4 Department of Paediatrics and Child Health, College of Health Sciences, Ladoke Akintola University of Technology, Osogbo, Nigeria

**Keywords:** Polycystic kidney disease, Newborn, Nigeria

## Abstract

Autosomal recessive polycystic kidney disease (ARPKD) is a rare genetic disorder but even rarer in Africans and it is one of the causes of nephropathies in childhood. Although isolated cases of adult PKD have been reported in Nigerians; to the best of our knowledge, this case is the first to be reported in the paediatric age group in Nigeria. A case of autosomal recessive polycystic kidney disease presenting with severe perinatal asphyxia and severe respiratory distressis here by presented. Fetal ultrasonography during the pregnancy missed the diagnosis. The difficulty in making diagnosis and management is discussed. Autopsy helped to unravel the diagnosis in this case report.

## Introduction

Polycystic kidney disease can be inherited as an autosomal recessive (ARPKD) or autosomal dominant trait (ADPKD). Autosomal recessive polycystic kidney disease (ARPKD) is a rare genetic disorder but even rarer in Africans and it is one of the causes of nephropathies in childhood 1. It occurs with an estimated incidence of 1 in 20,000 live births [1]. Moreso, autosomal recessive polycystic kidney disease (ARPKD) very rarely exists in isolation; it often presents as Caroli's syndrome when Caroli disease is associated [2]. Both Caroli disease and Caroli syndrome are rare congenital disorders of the intrahepatic bile ducts characterized by dilatation of the intrahepatic biliary tree [3,4]. In Caroli syndrome, the malformations of small bile ducts and congenital hepatic fibrosis are often associated with autosomal recessive polycystic kidney disease (ARPKD). Caroli syndrome is generally inherited in an autosomal recessive manner similar to ARKPD [5-7]. The autosomal recessive pattern of inheritance is currently believed to be caused by a single gene with linkage to locus 6p21. This gene has been termed polycystic kidney and hepatic disease gene 1(PKHD 1) [8]. A frequency of 1 in 70 of carriers of the PKHD 1 gene exists in the general population [9]. The PKHD 1 gene manifests with widely varying severity of both the renal and the hepatic disease [10]. ARPKD is rare in Nigeria and the present case had many uncommon presentations hence the case presentation.

## Patient and observation

**Figure 1:**
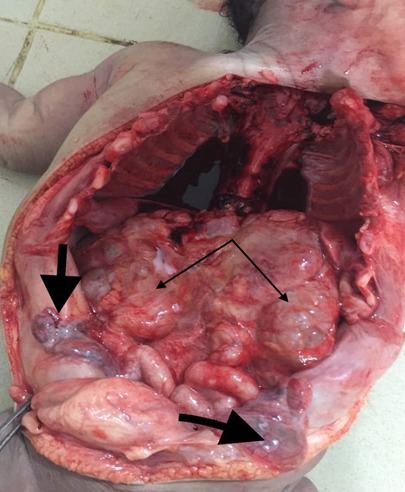
Showing enlarged kidneys (light arrows) and bilateral intra abdominal testes

**Figure 2:**
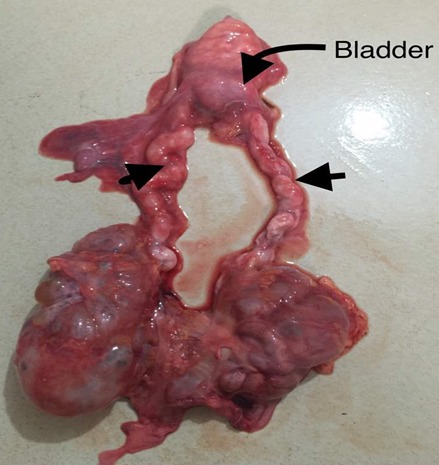
Showing dilated and tortuous ureters

**Figure 3:**
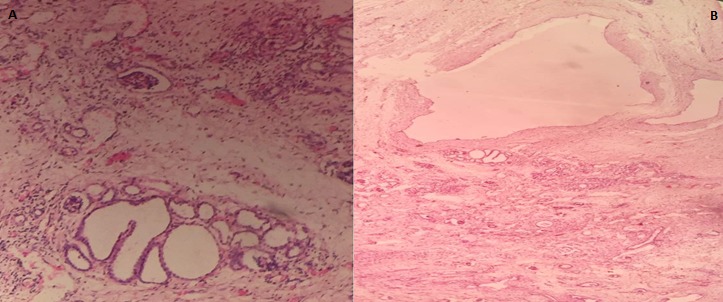
(A) showing variably sized cysts and poorly formed glomerular structures (H&E x 400); (B) showing variably sized cysts and poorly formed glomerular structures (H&E x 400)

A 9 hour old term male neonate was rushed into the neonatal unit of our facility with severe respiratory distress and gasping breathing. He was delivered in a private hospital by emergency caesarean section on account of fetal distress and breech presentation. The details of immediate postnatal events and resuscitation measures could not be obtained because baby was brought in by maternal grandmother while the mother was still at the referral hospital. Pregnancy history was said not be adversely eventful and all ultrasound scan during the prenancy was reportedly normal. Patient was the 2^nd^ child of parents in a monogamous setting. Elder sibling is alive and well. There were no known cases of renal or other congenital abnormalities in any members of the family including the first sibling. At presentation, he had gasping respiration and was resuscitated. However, he remained acutely ill looking with severe respiratory distress, centrally and peripherally cyanosed. Weight was 4.2kg while the length was 56cm. He was unconscious, floppy and all the primitive reflexes were absent. Abdominal examination showed hepatomegaly and bilateral massive nephromegaly. Both testes were neither palpable in the scrotal sac nor along the inguinal canal. The anus was patent. Respiratory system examination showed poor intensity of the breath sounds. Cardiovascular examinations revealed regular, small volume pulses and normal heart sounds. A clinical diagnosis of respiratory distress probably secondary to severe perinatal asphyxia, and to keep-in-view congenital anomaly of the genitourinary tract (PKD or Caroli syndrome) was made. He had respiratory support with intranasal oxygen and bubble CPAP; intravenous fluids, antibiotics. A urethral catheter passed after considerable difficulty to monitor urinary output drained no urine after 1 hour of admission even after fluid challenge with normal saline to improve renal perfusion. Further investigations like abdominal radiograph and USS could not be done due to the patient's unstable clinical condition and the unavailability of bedside facilities/ mobile equipment to do it. Patient desaturation was persistent as the SPO2 ranged between 40% and 52% on 100% humidified oxygen and CPAP. Clinical condition continued to deteriorate andhe was eventually certified dead at 17 hours of life. No urine was recorded. The main autopsy findings are as Figure 1 which is Photograph showing enlarged kidneys (light arrows) and bilateral intra abdominal testes. The genitourinary system showed grossly enlarged kidneys, with dilated and tortuous ureters as in Figure 2. The right kidney measured 8cm x 5cm x 2 cm while the left one measured 7cm x 5cm x 2cm. Cut sections of the kidneys showed numerous, variably sized cysts, these cysts were seen on light microscopy to be lined by low cuboidal to flattened epithelium. Also seen were poorly formed glomerular structures as shown in Figure 3A and Figure 3B which are Photomicrographs showing variably sized cysts and poorly formed glomerular structures (H&E x 400). Histology further suggested infantile polycystic kidney disease The bladder was distended with some urine and the wall was thickened but a posterior urethral valve was not demonstrated. Both testes were small, shrunken and found in the posterior abdominal wall as in Figure 1. The lungs were small, dull in appearance and firm; almost all portions were airless and sank in water. There was also hepatomegaly. The liver showed with features of generalized congestion; however, neither periportal nor hepatic fibrosis was demonstrable histologically and no ductal dilatation. Diagnosis of Autosomal recessive polycystic kidney disease (ARPKD) was therefore made at autopsy.

## Discussion

The present case is an example of isolated autosomal recessive polycystic kidney disease; in view of the absence of similar renal disease in neither previous sibling nor any positive family history as in autosomal dominant form polycystic kidney disease. It is also not fit for the diagnosis of Caroli syndrome because of absence of hepatic fibrosis. Diagnosis of the present case was difficult until autopsy was done. For instance, the ultrasound during pregnancy was reported normal with adequate amniotic fluid. Why the present case had adequate liquor despite being a very severe form with grave effect is not clear. Infants with severe renal involvement in utero usually have oligohydramnios-induced pulmonary hypoplasia and often die in the immediate postnatal period [1]. The lungs were small, dull in appearance and firm; almost all portions were airless and sank in water. This may be evidence of pulmonary hypoplasia which made the patient to present as severe birth asphyxia. There was only minimal respiratory effort with gasping breathing despite ventilatory support. Even though, there was no demonstrable ultrasound diagnosis of oligohydraminos, the patient had signs of genitourinary outlet obstruction with distended bladder containing urine and the wall was thickened but a posterior urethral valve was not demonstrated. Perhaps, the oligohydraminous was missed. Bilateral renal agenesis, bilateral multicystic or polycystic kidneys and renal dysplasia area consequence of early-onset bladder outlet obstruction from either posterior urethral valves or urethral stenosis are lethal abnormalities in the neonatal period due to pulmonary hypoplasia [1-4]. Pulmonary hypoplasia is known to be common in the severe form of ARKPD [4,5]. The present case showed a severe form of ARKPD. During fetal development, the kidney is a major source of proline production which aids in the formation of collagen and mesenchyme in the lung. The absence of this vital protein that in part explain the severe pulmonary hypoplasia seen in polycystic kidney disease, renal agenesis or renal dysplasias [11,12]. The main diagnostic modality for neonatal renal cystic diseases is ultrasound imaging and most cases of neonatal renal cystic diseases (autosomal dominant PKD, autosomal recessive PKD and multicystic dysplastic PKD) are detected during prenatal USS screening [13]. This case however was not detected on USS underscoring the importance of improving diagnostic skills at the level of peripheral centres. Neonatal ARPKD can result in severe morbidity and grave prognosis. The factors that modulate gene expression of severity are yet to be defined and there remains therefore, a widespread pessimism about prognosis for ARPKD patients [13]. If the patient survives the neonatal period, current therapies include the management of arterial hypertension, dietary modifications such as a low protein diet, aggressive ventilatory support and renal replacement therapy (including pre-emptive nephrectomy, dialysis and transplantation) [14]. This may help minimize early infant mortality and give affected infants a favourable outcome. Unfortunately, the patient in the present case died before any useful and significant intervention could be done.

## Conclusion

Neonatal polycystic kidney disease may not be as rare as previously reported particularly in blacks. It is probably underdiagnosed especially where the appropriate skill for fetal ultrasonogram diagnosis needs improvement. Availability of good antenatal care and early ultrasound detection will improve the detection. Referral of suspected case before delivery to better equipped tertiary health facilities for early diagnosis and intervention can provide the advanced care that affected neonates require. This may also assist data collection on the prevalence, presentations and possible modalities of treatment that will invariably improve the prognosis and present neonatal health indices.

## Competing interests

The authors declare no competing interests.
